# In Vitro Antioxidant Potential and Effect of a Glutathione-Enhancer Dietary Supplement on Selected Rat Liver Cytochrome P450 Enzyme Activity

**DOI:** 10.1155/2018/7462839

**Published:** 2018-05-24

**Authors:** Benoit B. N'guessan, Seth K. Amponsah, George J. Dugbartey, Kwabena D. Awuah, Eunice Dotse, Abigail Aning, Kennedy K. E. Kukuia, Isaac J. Asiedu-Gyekye, Regina Appiah-Opong

**Affiliations:** ^1^Department of Pharmacology and Toxicology, School of Pharmacy, College of Health Sciences, University of Ghana, Ghana; ^2^Department of Clinical Pathology, Noguchi Memorial Institute for Medical Research, College of Health Sciences, University of Ghana, Ghana

## Abstract

**Background:**

There is considerable evidence that many people take dietary supplements including those of herbal origin as an alternative therapy to improve their health. One such supplement, with an amalgam of constituents, is CellGevity®. However, the effect of this dietary supplement on drug-metabolizing enzymes is poorly understood, as it has not been studied extensively. Therefore, we investigated the effect of CellGevity dietary supplement on selected rat liver microsomal cytochrome P450 (CYP) enzymes, the most common drug-metabolizing enzymes. We also determined the total antioxidant potential of this dietary supplement* in vitro*.

**Methods:**

To determine the antioxidant potential of CellGevity dietary supplement, 2,2-diphenyl-2-picryl-hydrazyl (DPPH), total phenolic, and flavonoid assays were used after initial preparation of a solution form of the supplement (low dose, LD; 4 mg/kg and high dose, HD; 8 mg/kg). Rats received oral administration of these doses of the supplement for 7 days, after which the effect of the supplement on selected liver CYP enzymes was assessed using probe substrates and spectroscopic and high-performance liquid chromatographic methods. Rats which received daily administration of 80 mg/kg of phenobarbitone and distilled water served as positive and negative controls, respectively.

**Results:**

The IC_50_ value of the supplement 0.34 ± 0.07 mg/ml compared to 0.076 ± 0.03 mg/ml of the BHT (positive control). The total phenolic content of the supplement at a concentration of 2.5 mg/ml was 34.97 g gallic acid equivalent (GAE)/100 g while its total flavonoid content at a concentration of 2.5 mg/ml was 6 g quercetin equivalent (QE)/100 g. The supplement significantly inhibited rat CYP2B1/2B2 (LDT 92.4%; HDT 100%), CYP3A4 (LDT 81.2%; HDT 71.7%), and CYP2C9 (LDT 21.7%; HDT 28.5%) while it had no significant inhibitory effect on CYPs 1A1/1A2, CYP1A2, and CYP2D6.

**Conclusion:**

CellGevity dietary supplement possesses moderate antioxidant activity* in vitro* and has an inhibitory effect on selected rat liver CYP enzymes, suggesting its potential interaction with drugs metabolized by CYP enzymes.

## 1. Introduction

Noncommunicable diseases (NCDs) such as cardiomyopathies, asthma, diabetes mellitus, and cancer are the most common causes of death globally, with a higher percentage of premature deaths happening in developing nations than in developed nations [1]. This highlights the crucial need for simple and effective preventive strategies and treatments to reduce the current inequities within and among countries. At least half of these NCDs-related deaths are caused by common risk factors including malnutrition, a condition that represents a critical public health concern [2, 3]. Malnutrition occurs when the nutritional needs for growth (protein and calories) are not met within the context of either undernutrition or overnutrition and lead to deficiencies of essential micronutrients, with detrimental and sometimes irreversible effects [4].

The use of alternative therapies, in the form of dietary supplements, is becoming very common throughout the world, as many people nowadays are adopting a variety of lifestyle habits that contribute to healthy living [5]. Dietary supplements comprise a wide range of products intended for ingestion to meet essential nutritional needs. They may be individual components or combinations of vitamins, minerals, amino acids, or herbal products and have intermediate form between foods and drugs [6]. Thus, they possess both food and drug characteristics, a number of them being more food-like or drug-like, depending on their nature.

Dietary supplements are essential when nutritional needs are not covered by diet alone; however, the use of dietary supplementation when nutritional sufficiency has already been achieved remains controversial, as possible toxic effects of excessive intake have been reported for some micronutrients such as *β*-carotene and vitamin E [7, 8]. Whereas the quest for improved health with dietary supplements is commendable, there is a paucity of scientific data on some of the purported therapeutic efficacies of these dietary supplements.

Dietary supplements, including those of herbal origin, are known to alter the pharmacokinetics of concomitantly administered conventional drugs [9]. These supplements (or their constituents) often induce or inhibit drug-metabolizing enzymes such as cytochrome P450 (CYP), which play significant roles in phase I biotransformation reactions, converting lipophilic agents into hydrophilic metabolites and thereby facilitating excretion [10]. A typical example of a dietary supplement (herb) that modulates the activities of CYP enzymes is St. John's wort* (Hypericum perforatum)* [11, 12].

A number of dietary supplements currently available on the market have been reported to replenish levels of reduced glutathione (GSH), the most abundant naturally occurring antioxidant in the body [13]. Despite a scarcity of available scientific evidence, these GSH-enhancer dietary supplements are purported to play a potential role in the prevention of NCDs, especially those mediated by free radicals and characterized by depleted stores of tissue GSH [14]. One of such supplements, CellGevity, contains the GSH-precursor molecule, riboceine (D-ribose-L-cysteine), which has been reported to effectively deliver cysteine into the cell and enhance GSH level [15]. Riboceine has been shown to be significantly more effective than other glutathione enhancers [16], hence, the rationale for the choice of this dietary supplement in the present study.

In addition to riboceine, CellGevity contains an amalgam of constituents comprising turmeric root extract (curcumin), resveratrol, aloe extract, milk thistle, quercetin, broccoli seed extract, alpha lipoic acid, grape seed extract, vitamin C, selenomethionine, cordyceps, and piperine. Some of these constituents are known as inducers and/or inhibitors of CYP enzymes. Curcumin and resveratrol, for example, are potent inhibitors of CYP enzymes [17–19], while* aloe vera* induces CYP reductase and some Phase II enzymes [20].

Given the reported cases of induction and/or inhibition of CYP enzymes by some of its constituents and the potential supplement-drug interaction that may ensue, the present study investigates the effect of CellGevity dietary supplement on the activities of selected rat liver microsomal CYP enzymes and evaluates its total antioxidant potential.

## 2. Materials and Methods

*Ethical Statement*. All animal work was conducted according to the guidelines of the National Institute of Health for the Care of Laboratory Animals [21] and was approved by the Scientific and Technical Committee of Noguchi Memorial Institute for Medical Research, University of Ghana. 

*Experimental Animals*. Prior to experiment, 20 male Sprague Dawley rats weighing 300 ± 50 g (≥8 weeks old) from the Animal Experimentation Unit, Center for Plant Medicine Research, Mampong-Akuapem, Ghana, were fed ad libitum using standard animal lab pellet (Sankofa Flour and Feeds, Accra, Ghana) and were housed in 4 groups of 5 animals per cage under standard laboratory conditions (25 ± 1°C ambient temperature, 60–70% relative humidity, and 12:12 h light : dark cycle) to acclimatize to the laboratory condition for 7 days. 

*Treatment Groups*. Following acclimatization, rats were randomly assigned to one of the four experimental groups, being low dose supplement treatment (LDT; *n* = 5), high dose supplement treatment (HDT; *n* = 5), positive control (PC; *n* = 5), and negative control (NC; *n* = 5). The LDT group received a daily dose of 4 mg/kg of the supplement while the HDT group received 8 mg/kg. The PC group received a daily administration of 80 mg/kg of phenobarbitone, whereas the NC group was given distilled water daily. Each group received their respective treatment via oral route for 7 days. Following 7 days of treatment, animals were sacrificed by injection of an overdose of sodium pentobarbital intraperitoneally, and liver samples were harvested and snap-frozen in liquid nitrogen and stored at −80°C until further analysis.

### 2.1. Antioxidant Assays

*2,2-Diphenyl-2-Picryl-Hydrazyl (DPPH) Assay*. The DPPH method used was a modification of one reported by Blois [22]. Briefly, 20 mg of the supplement (CellGevity powder; Max International, Ghana) was dissolved in 1.0 ml of dimethyl sulfoxide (DMSO; Sigma Aldrich, USA) to obtain a stock solution of 20 mg/ml. Twofold serial dilutions of the stock were made to obtain concentrations of 10, 5, 2.5, 1.25, 0.625, and 0.3125 mg/ml. Twofold serial dilutions of the positive control, butylated hydroxyl toluene (BHT; St. Louis, MO, USA), were made to obtain concentrations of 0.5, 0.25, 0.125, 0.0625, 0.03125, and 0.015625 mg/ml. One hundred microliters of each of the samples and BHT dilutions was pipetted separately in triplicate into 96-well plates. A volume of 100 *μ*L of 0.5 mM DPPH solution (Steinheim, Germany) was then added to each of the wells to obtain a total volume of 200 *μ*L. The plates were incubated in the dark at room temperature for 20 minutes and absorbance was read at a wavelength of 517 nm. 

*Total Phenolic Assay*. The assay used to estimate total phenols in the supplement was a modification of one reported by Marinova et al. [23]. Briefly, a stock solution of the supplement was prepared by dissolving 20 mg of the sample in 1.0 ml of DMSO. Twofold serial dilutions of this stock were made to obtain concentrations of 10.0, 5.0, 2.5, and 1.25 mg/ml. The standard was prepared by dissolving 1.0 mg of gallic acid (generously provided by the Department of Nutrition and Food Science, University of Ghana) in 10% absolute ethanol. Twofold serial dilutions were made to obtain concentrations of 0.5, 0.25, 0.125, 0.0625, 0.03125, and 0.015625 mg/ml. One hundred microliters of each of the sample dilutions and the standard was pipetted separately in triplicate into 96-well plates. A volume of 100 *μ*L of Folin-Ciocalteu reagent (Buchs, Switzerland) was then added to each well followed by 200 *μ*L of sodium bicarbonate solution (0.2 g/ml) to obtain a total volume of 400 *μ*L. The plates were incubated at room temperature for 120 minutes and absorbance read at a wavelength of 650 nm. 

*Total Flavonoid Assay*. The total flavonoid assay used was one adapted from Ordoñez et al. [24]. Briefly, a stock solution of the supplement was prepared and diluted to obtain concentrations of 10.0, 5.0, 2.5, and 1.25 mg/ml. Quercetin standard (Buchs, Switzerland) was prepared and diluted to obtain concentrations of 0.1, 0.05, 0.025, 0.0125, 0.00625, 0.003125, and 0.0015625 mg/ml. One hundred microliters of each of the sample dilutions and the standard was pipetted separately in triplicate into 96-well plates. A volume of 100 *μ*L of aluminum chloride solution (2% w/v; Sigma Aldrich, USA) was added to each of the wells to obtain a final volume of 200 *μ*L per well. The plates were then incubated at room temperature for 20 minutes after which absorbance was read at a wavelength of 415 nm.

### 2.2. Rat Liver CYP Enzyme Induction/Inhibition Assays

*Preparation of Microsomal Fractions and Protein Level Determination*. Liver samples weighing 7.82 g were homogenized separately with two volumes of potassium phosphate buffer (pH 7.4) in a mortar with pestle. The homogenate was centrifuged at 4,500 rpm for 20 minutes at 4°C and the supernatant collected. Next, the supernatant was further centrifuged at 40,000 rpm for 60 minutes at 4°C with an ultra-centrifuge (Beckman Avanti J-25, USA). Following ultra-centrifugation, the resultant supernatant (cytosol) was separated from the pellet (microsomes). The microsomes obtained were then homogenized in potassium phosphate buffer (pH 7.4) to form a solution. Fourfold serial dilutions were carried out on the microsomal solutions using potassium phosphate buffer. Serial dilutions (2-fold, 6 dilutions) were also made with a protein standard, bovine serum albumin (BSA; St. Louis, MO, USA). Ten microliters of the BSA and 200 *μ*L of Biorad reagent (Bio-Rad Laboratories Inc., USA) was added to each microsomal dilution in a 96-well plate and incubated at room temperature for 5 minutes, and absorbance was read at a wavelength of 590 nm.

*CYP1A1/1A2-Ethoxyresorufin O-Deethylase (EROD), CYP1A2-Methoxyresorufin O-Demethylase (MROD), CYP3A4-BenzyloxyresorufinO-Debenzylase (BROD), and CYP2B1/2B2-Pentoxyresorufin O-Depentylase (PROD) Assays*. Inhibition of CYP 1A1/1A2, 1A2, 3A4, and 2B1/2B2 enzymes by the supplement was determined using fluorimetric assays similar to ones described by Appiah-Opong et al. [17] and Umegaki et al. [25] but with slight modification. Briefly, 70 *μ*L of potassium phosphate buffer (pH 7.4) was pipetted in triplicate into a 96-well plate followed by addition of 10 *μ*L of each substrate (ethoxyresorufin, methoxyresorufin, benzyloxyresorufin, and pentoxyresorufin purchased from St. Louis, MO, USA). Next, 10 *μ*L of the rat liver microsomal fraction obtained from each treatment group was added and incubated at 37°C for 5 min. Ten microliters (100 *μ*M) of nicotinamide adenine dinucleotide phosphate (NADPH; St. Louis, MO, USA) was added to each of the wells and incubated at 37°C for 10, 20, and 30 min (for CYPs 1A1/1A2 and 1A2, 2B1/2B2 and 3A4, respectively). A volume of 40 *μ*L of stopping solution (20% 0.5 M Tris and 80% acetonitrile) was added and the plate gently was shaken. Fluorescence was read at specific wavelengths at 586 nm. 

*CYP2D6-Dextromethorphan O-Demethylation Assay*. The effect of the supplement on dextromethorphan O-demethylation by CYP2D6 was assayed as described by Appiah-Opong et al. [17]. Briefly, 350 *μ*L of potassium phosphate buffer (pH 7.4) was pipetted into Eppendorf tubes in triplicate. Fifty microliters of 0.25 mM dextromethorphan (Milan, Italy) was added followed by 50 *μ*L of microsomes obtained from each group. Preincubation was done at 37°C for 5 minutes in a water bath after which 50 *μ*L of NADPH solution (100 *μ*M) was added. Further incubation was done for 45 minutes, followed by the addition of 100 *μ*L of stopping solution (300 mM zinc sulphate heptahydrate). The mixture was centrifuged at 4,000 rpm for 15 min at room temperature, and the supernatant was collected in vials and analyzed using an isocratic HPLC method with a C18 column (150 mm × 4.6 mm, VP-ODS). The mobile phase consisted of 24% (v/v) acetonitrile and 0.1% (v/v) trimethylamine adjusted to pH 3.0 with perchloric acid. The carrier flow rate was 0.8 ml/min and peaks were monitored at wavelengths of 280 nm (excitation) and 310 nm (emission). 

*CYP2C9-Diclofenac Hydroxylation Assay*. The effect of the supplement on hydroxylation of diclofenac to 4-hydroxydiclofenac by CYP2C9 was determined as described by Appiah-Opong et al. [26]. Briefly, 350 *μ*L of potassium phosphate buffer (pH 7.4) was pipetted into Eppendorf tubes followed by 50 *μ*L of 0.05 mM diclofenac (Overrijse, Belgium). Next, 50 *μ*L of the microsomal fraction obtained from each treatment group was added (in triplicate) and preincubated at 37°C for 5 minutes in a water bath. A volume of 50 *μ*L of NADPH solution (100 *μ*M) was added to each tube and further incubated in the water bath at 37°C for 10 minutes. The reaction was terminated by addition of 200 *μ*L of stopping solution (ice-cold methanol) and the mixture was centrifuged at 12,000 rpm for 8 minutes at room temperature. The supernatants were collected in vials and analyzed using high-performance liquid chromatography (HPLC) [Agilent 1100 Series, Germany]. The HPLC conditions for the assay comprised an injection volume of 50 *μ*L, a flow rate of 0.8 ml/min, a C18 column (150 mm × 4.6 mm, VP-ODS), a temperature of 40°C, and a maximum pressure of 40 bar. A diode array served as the detector. Products formed were measured using an isocratic HPLC method. The mobile phase consisted of 60% of 20 mM potassium phosphate buffer (pH 7.4), 22.5% methanol, and 17.5% acetonitrile.

### 2.3. Statistical Analysis

All values are expressed as mean ± standard deviation (SD). Differences between groups were tested for significance using a One-Way ANOVA. *p* values < 0.05 were considered statistically significant. Significant differences were calculated with Bonferroni's Multiple Comparison Tests, and graphs were produced using Graph Pad Prism Software Version 7 (GraphPad Software, Inc., USA).

## 3. Results

### 3.1. Antioxidant Assays

**Figure 1:**
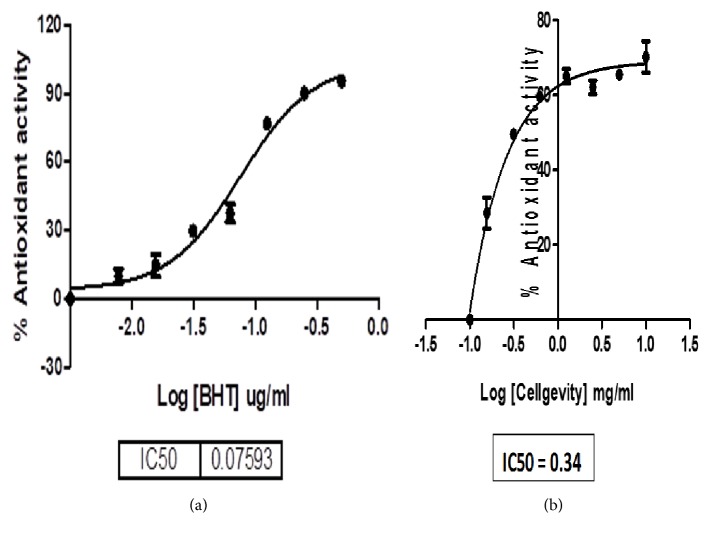
Concentration-response curves showing IC_50_ values for butylated hydroxytoluene (BHT; positive control (a)) and CellGevity dietary supplement (b).

To evaluate the antioxidant potential of CellGevity dietary supplement, DPPH, total phenolic, and flavonoid assays were used. The concentration of the supplement required to inhibit 50% of free radicals (IC_50_) was 0.34 ± 0.07 mg/ml compared to 0.076 ± 0.03 mg/ml of the BHT (positive control). The total phenolic content of the supplement at a concentration of 2.5 mg/ml was 34.97 g gallic acid equivalent (GAE)/100 g while its total flavonoid content at a concentration of 2.5 mg/ml was 6 g quercetin equivalent (QE)/100 g. Figures 1(a) and 1(b) show the IC_50_ values of CellGevity as compared to BHT.

### 3.2. CYP Enzyme Assays

In order to determine the effect of CellGevity dietary supplement on rat liver microsomal cytochrome P450 (CYP) enzyme activities, selected CYP assays were used. 

**Figure 2:**
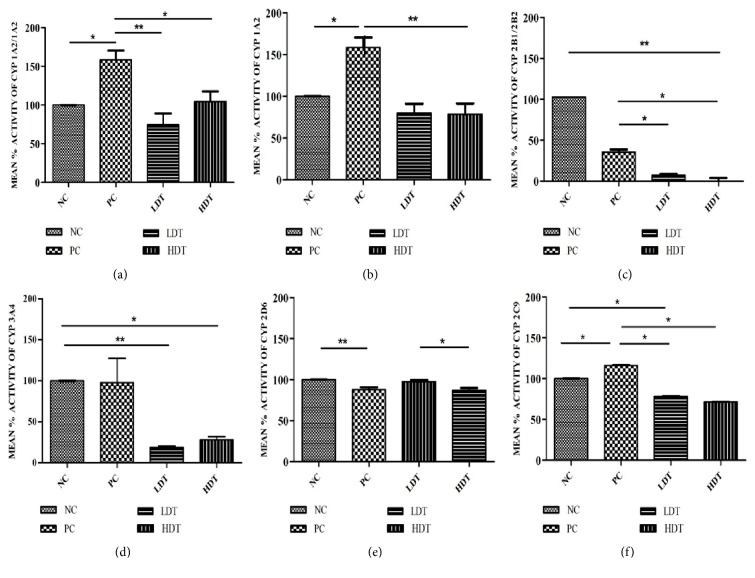
(a)* Effect of CellGevity dietary supplement on CYP1A1/1A2 activity in rat liver microsomes*. Negative control (NC; distilled water), positive control (PC; phenobarbitone 80 mg/kg), low dose treatment (LDT; 4 mg/kg supplement), and high dose treatment (HDT; 8 mg/kg supplement). Data represent mean ± standard deviations. *∗* and *∗∗* are values statistically different as indicated with *p* < 0.05 and *p* < 0.001, respectively. (b)* Effect of CellGevity dietary supplement on CYP1A2 activity in rat liver microsomes*. Negative control (NC; distilled water), positive control (PC; phenobarbitone 80 mg/kg), low dose treatment (LDT; 4 mg/kg supplement), and high dose treatment (HDT; 8 mg/kg supplement). Data represent mean ± standard deviations. *∗* and *∗∗* are values statistically different as indicated with *p* < 0.05 and *p* < 0.001, respectively. (c)* Effect of CellGevity dietary supplement on CYP2B1/2B2 activity in rat liver microsomes*. Negative control (NC; distilled water), positive control (PC; phenobarbitone 80 mg/kg), low dose treatment (LDT; 4 mg/kg supplement), and high dose treatment groups (HDT; 8 mg/kg supplement). Data represent mean ± standard deviations. *∗* and *∗∗* are values statistically different as indicated with *p* < 0.05 and *p* < 0.001, respectively. (d)* Effect of CellGevity dietary supplement on CYP3A4 activity in rat liver microsomes*. Negative control (NC; distilled water), positive control (PC; phenobarbitone 80 mg/kg), low dose treatment (LDT, 4 mg/kg supplement), and high dose treatment (HDT; 8 mg/kg supplement). Data represent mean ± standard deviations. *∗* and *∗∗* are values statistically different as indicated with *p* < 0.05 and *p* < 0.001, respectively. (e)* Effect of CellGevity dietary supplement on CYP2D6 activity in rat liver microsomes*. Negative control (NC; distilled water), positive control (PC; phenobarbitone 80 mg/kg), low dose treatment (LDT, 4 mg/kg supplement), and high dose treatment (HDT; 8 mg/kg supplement). Data represent mean ± standard deviations. *∗* and *∗∗* are values statistically different as indicated with *p* < 0.05 and *p* < 0.001, respectively. (f)* Effect of CellGevity dietary supplement on CYP2C9 in rat liver microsome*. Negative control (NC; distilled water), positive control (PC; phenobarbitone 80 mg/kg), low dose treatment (LDT, 4 mg/kg supplement), and high dose treatment (HDT; 8 mg/kg supplement). Charts represent mean ± standard deviations. *∗* are values statistically different as indicated with *p* < 0.05.

*CYP1A1/1A2 and CYP1A2 Assays*. There was no significant difference in the activity of CYP1A1/1A2 enzyme among NC, LDT, and HDT groups (Figure 2(a); *p* > 0.05). However, the CYP1A1/1A2 enzyme activity of these three groups markedly decreased compared to PC group (Figure 2(a); *p* < 0.05). A similar observation was made in CYP1A2 enzyme activity (Figure 2(b)). 

*CYP2B1/2B2 and CYP3A4 Assays*. Unlike CYP1A1/1A2 and CYP1A2 enzyme activities, which showed no significant difference between NC, LDT, and HDT groups in the microsomal fractions, the activity of the CYP2B1/2B2 enzyme in LDT and HDT groups markedly decreased compared to NC group (Figure 2(c); *p* < 0.001). A similar pattern was observed in Figure 2(d). However, whereas the PC group showed markedly reduced CYP2B1/2B2 enzyme activity compared to NC group in Figure 2(c) (*p* < 0.05), that in CYP3A4 in Figure 2(d) showed no difference in comparison with the NC group (*p* > 0.05). 

*CYP2D6 and CYP2C9 Assays*. CYP2D6 enzyme activity in LDT group increased significantly compared to HDT group (Figure 2(e); *p* < 0.05). In addition, NC group showed a markedly high CYP2D6 activity in comparison with PC group (Figure 2(e); *p* < 0.001). As seen in Figure 2(b), the activity of CYP2C9 followed a similar pattern in which no significant difference was observed between LDT and HDT groups while activity in NC group also markedly decreased (Figure 2(f); *p* < 0.05). 

*Overall Effect of the Supplement on Rat CYP Enzymes*. The overall effect of this supplement on selected CYP enzymes is summarized in Table 1. Inhibition of CYP enzyme activity by the supplement was not dose-dependent. The general trend of enzyme inhibition (highest to lowest) by both doses of the supplement was CYP2B1 > CYP3A4 > CYP2C9 > CYP1A1/1A2 > CYP1A2 > CYP2D6.

## 4. Discussion

In the current study, we evaluated the antioxidant potential of CellGevity dietary supplement, comprising an aggregate of ingredients, and the effect of this supplement on the activities of selected rat liver microsomal enzymes. This study focuses on CYP enzymes (one of the conserved entities among species) which are the main enzymes involved in numerous oxidative reactions and often play a critical role in the metabolism and pharmacokinetics of xenobiotics. It is well established that some rat CYP enzymes are closely related to those of humans. For example, CYP1A shows a strong conservation among species with an identity to human > 80% in rat (83 and 80%, respectively, for CYP1A1 and -1A2) [27, 28].

Some constituents of CellGevity dietary supplement, such as curcumin, resveratrol, milk thistle, quercetin, and piperine, are known inhibitors of CYP3A4 [17–19, 29–31]. Hence, it is not surprising that this isoform was one of the enzymes significantly inhibited by the dietary supplement. CYP3A4 is one the most abundant CYP isoforms in human liver and is involved in the biotransformation of the majority of drugs [32]. However, some discrepancies between rats and human CYP3A4 isoforms, in the metabolism of drugs such as dihydropyridine calcium-channel blockers (e.g., nifedipine), have been reported, probably suggesting that rat is not a good model to study CYP3A4 induction [28, 33, 34]. Therefore, data from the current study, suggesting that CellGevity dietary supplement could alter the metabolism of some drugs that serve as human CYP3A4 substrates, should be interpreted cautiously.

Our study also showed that CellGevity dietary supplement significantly inhibited rat CYP2B1/2B2. Curcumin, one of the constituents of the supplement, is a less potent inhibitor of rat CYP2B1/2B2 compared to CYP1A1/1A2 enzyme [35]. This earlier report contradicts our finding, as we observed a significant inhibitory effect of the dietary supplement on CYP2B1/2B2 enzyme activity but not on the activities of CYP1A1/1A2 and CYP1A2 enzymes. As the dietary supplement has several constituents that affect CYP enzyme activity, it is possible that these refuting observations could be due to the synergistic inhibitory action of other constituents on CYP2B1/2B2 activity besides curcumin. It is important to note that the CYP2B subfamily is more abundant in rodents than in humans. In humans, however, the orthologous form of CYP2B1/2B2 is CYP2B6 [36]. Appiah-Opong et al. [17] reported that curcumin inhibited the human CYP2B6 enzyme, which is consistent with our observation in rats. This inhibitory effect on CYP2B activity suggests potential interaction when CellGevity dietary supplement is coadministered with drugs metabolized by this subfamily of CYP enzymes.

Another CYP enzyme, significantly inhibited by CellGevity dietary supplement, was CYP2C9. Although the effect of individual constituents of this dietary supplement on CYP enzyme activity was not investigated in our study, rodent and human microsome studies have shown that resveratrol, a constituent of this dietary supplement, is a potent inhibitor of CYP2C9 [37, 38]. A diet containing 0.5% w/w resveratrol fed to mice for 12 weeks was found to enhance the anticoagulant activity of warfarin, suggesting possible inhibition of CYP2C9 [37]. Using losartan as a probe drug, a daily dose of 1.0 g of resveratrol administered for 4 weeks was found to inhibit human CYP2C9 by 2.71‐fold [38]. Previous reports also suggest that curcumin is a potent inhibitor of human recombinant CYP2C9 [17]. Additionally, two flavonolignans from milk thistle (another constituent of this dietary supplement) were found to inhibit human CYP2C9-mediated warfarin metabolism [39]. These pieces of evidence suggest that CellGevity dietary supplement could modulate human CYP2C9 enzyme activity.

In the current study, the EC_50_ value of CellGevity dietary supplement was 0.34 ± 0.07 mg/ml compared to 0.076 ± 0.03 mg/ml of the BHT. The total phenolic content of the supplement at a concentration of 2.5 mg/ml was 34.97 g gallic acid equivalent (GAE)/100 g while its total flavonoid content at a concentration of 2.5 mg/ml was 6 g quercetin equivalent (QE)/100 g. This antioxidant potential is moderately high compared to a related study where the authors reported a synergistic antioxidant activity of a green tea of herbal origin determined by an EC_50_ value of 33.5 mg/ml, a total phenolic, and flavonoid content of 2.5 g GAE/10 g and 1.2 g QE/10 g, respectively [40]. Furthermore, there have been studies including ours in which authors reported antioxidant potential of dietary supplements using* in vitro* assays [41]. However, a constituent of CellGevity dietary supplement, riboceine, is a prodrug which requires bioactivation* in vivo*. Once in circulation, riboceine is metabolized into cysteine and ribose, which are transported into cells [15]. It is noteworthy that cysteine is a substrate for GSH synthesis in the liver and other organs [42], suggesting that CellGevity dietary supplement activates GSH pathway and possibly other endogenous antioxidants pathways, thereby bolstering the endogenous antioxidant defense system.

## 5. Conclusion

In conclusion, this study reports that CellGevity dietary supplement possesses antioxidant property* in vitro* and also inhibits activities of rat liver CYP2B1, CYP3A4, and CYP2C9 enzymes. Inhibition of these selected CYP enzymes by this dietary supplement suggests the possibility that CellGevity dietary supplement may contribute to supplement (herb-) drug interactions in humans.

## Figures and Tables

**Table 1:** A summary of the effect of the supplement on rat CYP enzymes.

CYP isoform	Assay	Effect of supplement on CYP activity
CYP 1A1/1A2	EROD	No significant decrease in enzyme activity
CYP 1A2	MROD	No significant decrease in enzyme activity
CYP 2B1/2B2	PROD	Significant decrease in enzyme activity (*p* < 0.001; LDT and HDT)
CYP 2C9	Diclofenac hydroxylation	Significant decrease in enzyme activity (*p* < 0.05; LDT)
CYP 2D6	Dextromethorphan O-demethylation	No significant decrease in enzyme activity
CYP 3A4	BROD	Significant decrease in enzyme activity (LDT: *p* < 0.001; HDT: *p* < 0.05)

## Data Availability

The data used to support the findings of this study are available from the corresponding author upon request.

## References

[1] World Health Organization World health statistics 2014. http://apps.who.int/iris/bitstream/10665/44844/1/9789241564441_eng.pdf.

[2] Beaglehole R., Bonita R., Alleyne G. (2011). UN high-level meeting on non-communicable diseases: addressing four questions. *The Lancet*.

[3] International Food Policy Research Institute 2014 global nutrition report: actions and accountability to accelerate the world’s progress on nutrition. http://ebrary.ifpri.org/utils/getfile/collection/p15738coll2/id/128484/filename/128695.pdf.

[4] World Health Organization The global prevalence of anaemia in 2011. http://apps.who.int/iris/bitstream/10665/177094/1/9789241564960_eng.pdf.

[5] Pitetti R., Singh S., Hornyak D., Garcia S. E., Herr S. (2001). Complementary and alternative medicine use in children. *Pediatric Emergency Care*.

[6] Food and Drug Administration (FDA) (2016). *Dietary Supplements: What You Need to Know*.

[7] Bjelakovic G., Nikolova D., Gluud L. L., Simonetti R. G., Gluud C. (2012). Antioxidant supplements for prevention of mortality in healthy participants and patients with various diseases. *Cochrane Database of Systematic Reviews*.

[8] Rautiainen S., Manson J. E., Lichtenstein A. H., Sesso H. D. (2016). Dietary supplements and disease prevention—a global overview. *Nature Reviews Endocrinology*.

[9] Brantley S. J., Argikar A. A., Lin Y. S., Nagar S., Paine M. F. (2014). Herb-drug interactions: challenges and opportunities for improved predictions. *Drug Metabolism and Disposition*.

[10] Cederbaum A. I. (2015). Molecular mechanisms of the microsomal mixed function oxidases and biological and pathological implications. *Redox Biology*.

[11] Miller L. G. (1998). Herbal medicinals: selected clinical considerations focusing on known or potential drug-herb interactions. *JAMA Internal Medicine*.

[12] Wang Z., Gorski J., Hamman M., Huang S., Lesko L., Hall S. (2001). The effects of St John's wort (Hypericum perforatum) on human cytochrome P450 activity. *Clinical Pharmacology & Therapeutics*.

[13] Bray T. M., Taylor C. G. (1994). Enhancement of tissue glutathione for antioxidant and immune functions in malnutrition. *Biochemical Pharmacology*.

[14] Fang Y.-Z., Yang S., Wu G. (2002). Free radicals, antioxidants, and nutrition. *Nutrition Journal *.

[15] Roberts J. C., Charyulu R. L., Zera R. T., Nagasawa H. T. (1992). Protection Against Acetaminophen Hepatotoxicity by Ribose-Cysteine (RibCys). *Pharmacology & Toxicology*.

[16] Oz H. S., Chen T. S., Nagasawa H. (2007). Comparative efficacies of 2 cysteine prodrugs and a glutathione delivery agent in a colitis model. *Translational Research*.

[17] Appiah-Opong R., Commandeur J. N. M., van Vugt-Lussenburg B., Vermeulen N. P. E. (2007). Inhibition of human recombinant cytochrome P450s by curcumin and curcumin decomposition products. *Toxicology*.

[18] Chan W. K., Delucchi A. B. (2000). Resveratrol, a red wine constituent, is a mechanism-based inactivator of cytochrome P450 3A4. *Life Sciences*.

[19] Piver B., Berthou F., Dreano Y., Lucas D. (2001). Inhibition of CYP3A, CYP1A and CYP2E1 activities by resveratrol and other non volatile red wine components. *Toxicology Letters*.

[20] Singh R. P., Dhanalakshmi S., Rao A. R. (2000). Chemomodulatory action of Aloe vera on the profiles of enzymes associated with carcinogen metabolism and antioxidant status regulation in mice. *Phytomedicine*.

[21] National Institutes of Health, “Memorandum of understanding between the office of Laboratory Animal Welfare, National Institutes of Health, US Department of Health and Human Services and the Office of Research Oversight and the Office of Research and Development, Veterans Health Administration, US Department of Veterans Affairs Concerning Laboratory Animal Welfare”. *Bethesda: Office of Extramural Research, NIH*. 2007. http://grants.nih.gov/grants/olaw/references/mou_olaw_va_2007_11.htm

[22] Blois M. S. (1958). Antioxidant determinations by the use of a stable free radical. *Nature*.

[23] Marinova D., Ribarova F., Atanassova M. (2005). Total phenolics and total flavonoids in Bulgarian fruits and vegetables. *Journal of the University of Chemical Technology and Metallurgy*.

[24] Ordoñez A. A. L., Gomez J. D., Vattuone M. A., Isla M. I. (2006). Antioxidant activities of *Sechium edule* (Jacq.) Swartz extracts. *Food Chemistry*.

[25] Umegaki K., Saito K., Kubota Y., Sanada H., Yamada K., Shinozuka K. (2002). Ginkgo biloba extract markedly induces pentoxyresorufin O-dealkylase activity in rats. *Japanese Journal of Pharmacology*.

[26] Appiah-Opong R., de Esch I., Commandeur J. N. M., Andarini M., Vermeulen N. P. E. (2008). Structure-activity relationships for the inhibition of recombinant human cytochromes P450 by curcumin analogues. *European Journal of Medicinal Chemistry*.

[27] Mugford C. A., Kedderis G. L. (1998). Sex-dependent metabolism of xenobiotics. *Drug Metabolism Reviews*.

[28] Martignoni M., Groothuis G. M., Kanter R. d. (2006). Species differences between mouse, rat, dog, monkey and human CYP-mediated drug metabolism, inhibition and induction. *Expert Opinion on Drug Metabolism & Toxicology*.

[29] Venkataramanan R., Ramachandran V., Komoroski B. J., Zhang S., Schiff P. L., Strom S. C. (2000). Milk thistle, a herbal supplement, decreases the activity of CYP3A4 and uridine diphosphoglucuronosyl transferase in human hepatocyte cultures. *Drug Metabolism and Disposition*.

[30] Umathe S. N., Dixit P. V., Kumar V., Bansod K. U., Wanjari M. M. (2008). Quercetin pretreatment increases the bioavailability of pioglitazone in rats: involvement of CYP3A inhibition. *Biochemical Pharmacology*.

[31] Bhardwaj R. K., Glaeser H., Becquemont L., Klotz U., Gupta S. K., Fromm M. F. (2002). Piperine, a major constituent of black pepper, inhibits human P-glycoprotein and CYP3A4. *The Journal of Pharmacology and Experimental Therapeutics*.

[32] Dresser G. K., Spence J. D., Bailey D. G. (2000). Pharmacokinetic-pharmacodynamic consequences and clinical relevance of cytochrome P450 3A4 inhibition. *Clinical Pharmacokinetics*.

[33] Guengerich F. P. (1997). Comparisons of catalytic selectivity of cytochrome P450 subfamily enzymes from different species. *Chemico-Biological Interactions*.

[34] Smith D. A. (1991). Species differences in metabolism and pharmacokinetics: Are we close to an understanding?. *Drug Metabolism Reviews*.

[35] Oetari S., Sudibyo M., Commandeur J. N. M., Samhoedi R., Vermeulen N. P. E. (1996). Effects of curcumin on cytochrome P450 and glutathione S-transferase activities in rat liver. *Biochemical Pharmacology*.

[36] Gonzalez F. J., Gelboin H. V. (1994). Role of human cytochromes p450 in the metabolic activation of chemical carcinogens and toxins. *Drug Metabolism Reviews*.

[37] Chiba T., Kimura Y., Suzuki S., Tatefuji T., Umegaki K. (2016). Trans-resveratrol enhances the anticoagulant activity of warfarin in a mouse model. *Journal of Atherosclerosis and Thrombosis*.

[38] Chow H.-H. S., Garland L. L., Hsu C.-H. (2010). Resveratrol modulates drug- and carcinogen-metabolizing enzymes in a healthy volunteer study. *Cancer Prevention Research*.

[39] Brantley S. J., Oberlies N. H., Kroll D. J., Paine M. F. (2010). Two flavonolignans from milk thistle (Silybum marianum) inhibit CYP2C9-mediated warfarin metabolism at clinically achievable concentrations. *The Journal of Pharmacology and Experimental Therapeutics*.

[40] Jain D., Pancholi S., Patel R. (2011). Synergistic antioxidant activity of green tea with some herbs. *Journal of Advanced Pharmaceutical Technology & Research*.

[41] Prior R. L., Wu X., Schaich K. (2005). Standardized methods for the determination of antioxidant capacity and phenolics in foods and dietary supplements. *Journal of Agricultural and Food Chemistry*.

[42] Saltman A. E. (2015). D-ribose-l-cysteine supplementation enhances wound healing in a rodent model. *The American Journal of Surgery*.

